# Magnetoliposomes as model for signal transmission

**DOI:** 10.1098/rsos.181108

**Published:** 2019-01-16

**Authors:** G. R. Barreto, C. Kawai, A. Tofanello, A. A. R. Neves, J. C. Araujo-Chaves, E. Belleti, A. J. C. Lanfredi, F. N. Crespilho, I. L. Nantes-Cardoso

**Affiliations:** 1 Center of Natural Sciences and Humanities (CCNH), Federal University of ABC (UFABC), Santo André, SP, Brazil; 2 Center for Engineering and Applied Social Sciences (CECS), Federal University of ABC (UFABC), Santo André, SP, Brazil; 3 São Carlos Institute of Chemistry, University of São Paulo (USP), Av. Trabalhador São-carlense, 400, São Carlos, São Paulo 13560-970, Brazil

**Keywords:** giant unilamellar vesicles, large unilamellar vesicles, nanoparticulated magnetite, signal transmission

## Abstract

Liposomes containing magnetic nanoparticles (magnetoliposomes) have been extensively explored for targeted drug delivery. However, the magnetic effect of nanoparticles movement is also an attractive choice for the conduction of signals in communication systems at the nanoscale level because of the simple manipulation and efficient control. Here, we propose a model for the transmission of electrical and luminous signals taking advantage of magnetophoresis. The study involved three steps. Firstly, magnetite was synthesized and incorporated into fusogenic large unilamellar vesicles (LUVs) previously associated with a fluorescent label. Secondly, the fluorescent magnetite-containing LUVs delivered their contents to the giant unilamellar vesicles (GUVs), which were corroborated by magnetophoresis and fluorescence microscopy. In the third step, magnetophoresis of magnetic vesicles was used for the conduction of the luminous signal from a capillary to an optical fibre connected to a fluorescence detector. Also, the magnetophoresis effects on subsequent transmission of the electrochemical signal were demonstrated using magnetite associated with CTAB micelles modified with ferrocene. We glimpse that these magnetic supramolecular systems can be applied in micro- and nanoscale communication systems.

## 1. Introduction

Self-organized structures and nanoparticles with different compositions, forms and sizes have been widely applied in catalysis, theranostic medicine, pharmacology development, nutrition, analytic and sensing techniques [1–5]. In the first category, liposomes formed by self-assembly of natural, synthetic and functionalized lipids are highlighted as useful structures for encapsulation, chemically directed targeting and peculiar microenvironments provided by interfaces that are applied in catalysis improvement and specificity [6–10]. Nanostructured magnetite (Fe_3_O_4_) combines the specific properties of nanostructures produced by the quantum confinement of electrons with magnetism [11]. The latter feature allows the controlled movement and targeting by the application of an external magnetic field. Liposomes are spherical structures comprising phospholipids organized as one or multiple concentric bilayers that are bounded by aqueous media at the outer and inner faces. According to the lamellar structure and size, liposomes are classified, respectively, as multilamellar vesicles (MLV) and unilamellar vesicles that, in turn, are small, large and giant vesicles (SUVs, LUVs and GUVs) [12]. Owing to a large number of applications in different areas, particularly in medical and pharmaceutical areas [3,8,13–16], liposomes have aroused the interest of many research groups over the past decades in the world [17].

Liposome suspensions constitute a heterogeneous system that provides at least three different microenvironments segregated from the bulk aqueous phase: the charged or neutral interface, the hydrophobic internal phase of the bilayer and internal aqueous compartment enclosed by one or multiple bilayers. Particularly, the challenge to promote delivery of a substance to a specific target has motivated the development and characterization of magnetic nanoparticles coated liposomes (magnetoliposomes) [10]. However, the study of properties of liposomes and magnetic liposomes has been predominantly concerned with drug delivery and membrane properties. We believe that the application of magnetoliposomes can be extended to bioelectronics, a field that involves the union of biological systems (biomolecules, organelles, cells) with electronics allowing the transduction of biological to electrical signals at the bioelectronic interface. In the development of bioelectronics, the challenge posed by the size disparity of transducers with biological systems led to increasing use of a variety of nanomaterials and the advent of nano-bioelectronics [18]. In the bioelectronic apparatus, the activity of an electrically contacted biomolecule-functionalized electrode, for application in catalysis and sensing, could be reversibly activated and deactivated by external signals. A bioelectronic device can also be controlled by using light and magnetism, in opto-bioelectronics and magneto-bioelectronics, respectively. The perspective for nanotechnology is beyond a multiplicity of nanoparticles and their static assembly. From now on, some studies have focused on communicating systems working by interacting nanoparts [18,19]. Communicating systems are applied in sensing, drug delivery, Boolean logical operations, among others [18,20]. In the communicating systems at the nanoscale, the guidance of nanoparticles movement is of particular importance.

**Figure 1.**
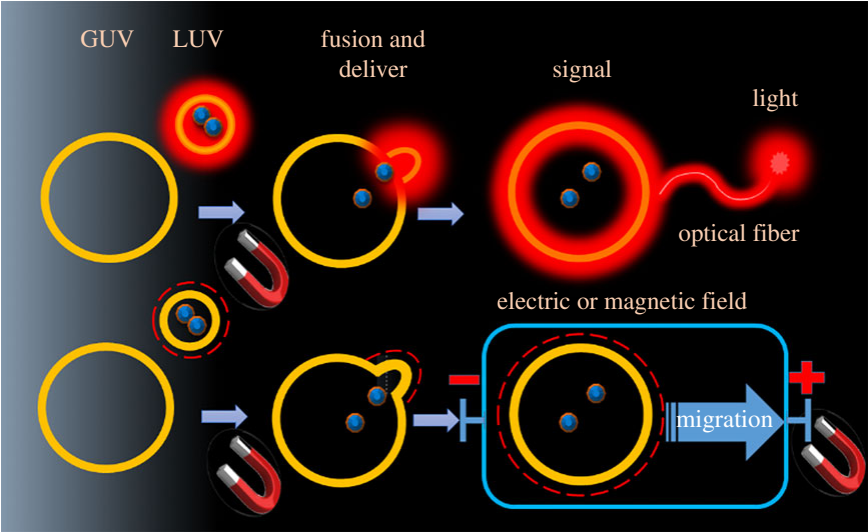
Versatility for the use of the delivery of particles and fluorophores to GUVs for the transmission of signals.

The functionalization can promote the natural movement of the nanoparticles in aqueous suspension driven by chemical binding affinity. However, this motion is usually irreversible and not deterministic. Where nanoparticles manipulation is concerned, optical tweezers are an option for capturing and conducting the movement of nanoparticles, but the manipulation is limited by the precise focusing requirements [21]. Magnetophoresis is an attractive alternative approach for controlling and manipulation of nanoparticles, mainly because the application of a magnetic field can be made by an arrangement of magnets in a very simple way, and no sophisticated and expensive equipment is required [22]. Since controlling nanoparticles in suspension can open several technological applications, here we propose a new approach to manipulate an LUV-mediated delivery of magnetite and fluorophores to GUVs [13,16,23,24]. Our strategy involved three major steps: (i) synthesis, characterization and incorporation of magnetite into fusogenic and fluorescent large unilamellar vesicles (LUVs); (ii) delivery of the magnetic and fluorescent content of LUVs to giant unilamellar vesicles (GUVs); (iii) the use of magnetophoresis of the magnetic vesicles for the conduction of the luminous and electric signal to the respective detectors. These steps are summarized in figure 1.

## 2. Material and methods

### 2.1. Materials

All solutions were prepared using reagents of high purity and ultra-pure water (Milli-Q, 18.2 MΩ cm^−1^). Ferric chloride 98% (anhydrous FeCl_3_), ferrous chloride tetrahydrate 98% (FeCl_2_.4H_2_O) and ammonium hydroxide 98% (NH_4_OH) were purchased from Vetec. Phosphatidylcholine (PC) from egg yolk, 1-palmitoyl-2-oleoyl-sn-glycero-3-phosphocholine (POPC), phosphoethanolamine (PE) from egg yolk, 1,2-dioleoyl-sn-glycero-3-phosphoethanolamine (DOPE), cardiolipin (CL) from bovine heart and tetraoleoyl cardiolipin (TOCL) were acquired from Avanti Polar Lipids, Inc., Alabaster, AL. Cetyltrimethylammonium bromide (CTAB), sucrose, β-d-glucose, sodium phosphate dihydrate (NaH_2_PO_4_) and sodium phosphate monohydrate (Na_2_HPO_4_), and the redox mediator ferrocene were acquired from Sigma-Aldrich, St Louis, MO. Chloroform (P.A.) from LabSynth, Diadema, SP, Brazil.

### 2.2. Preparation of LUVs and GUVs

Lipids were first dissolved in chloroform, which was evaporated with N_2_ gas. The lipid was hydrated with deionized water. Unilamellar liposomes were obtained by the sonication of hydrated lipid dispersions. For fluorescence measurements, minimal exposure of the lipids to light was ensured during the procedure [25]. GUVs of PC/PE/CL (50%/30%/20%, in weight, respectively) were grown with the electroformation method [26–29]. Briefly, 40 µl of a mixture of lipids 2.5 mM in chloroform solution was spread on the surfaces of two conductive glass plates (coated with fluorine doped tin oxide, FTO), which were then arranged with their conductive sides facing each other and separated by a 2 mm thick Teflon frame. This electro-swelling chamber was filled with 0.2 M sucrose solution and connected to an alternating power generator set at 1 V with a 10 Hz frequency for 2 h at 25°C. The vesicle solution was removed from the chamber and diluted approximately six times into 0.2 M glucose solution. In this condition, spherical GUVs were obtained with diameters in the range of 10 µm. The sugar optical asymmetry between the interior and the exterior of the vesicles created differences in density and the refractive index between the sucrose and glucose solutions; the vesicles were, therefore, stabilized by gravity at the bottom of the cavity, and had the best contrast when observed under phase-contrast microscopy. The observation of giant vesicles was performed under an inverted microscope, Zeiss Observe/A1 (Carl Zeiss, Jena, Germany), equipped with a 40× objective. Images were taken with an AxioCam R3 digital camera (Carl Zeiss). For these measurements, the vesicle solution was placed in a special chamber consisting of an 8 mm thick Teflon frame confined between two glass plates, through which observation was possible. For the fluorescence images with probed GUVs with MC540 (merocyanine 540), a filter set cube from Carl Zeiss (FS09) was used, with excitation at 450–490 nm.

### 2.3. Magnetite synthesis

A solution of 0.023 M ferric chloride was added to a 0.027 M ferrous chloride solution. The salts were stirred under an atmosphere of N_2_ and kept at room temperature while maintaining a molar ratio of Fe^2+^/Fe^3+^ of 1/2. Subsequently, 33.5 ml of concentrated NH_4_OH (27%) was added (pH between 10 and 11.8), and a black precipitate was formed immediately. The mixture was stirred (2000 r.p.m.) for 30 min using magnetic agitation. Finally, the product was decanted, washed three times and dried at 40°C for several hours [30].

### 2.4. Encapsulation of magnetite encapsulated by liposomes

Liposome-encapsulated nanoparticulated magnetite was prepared using PC/CL (80%/20% in weight, respectively). Chloroform solution (10 mM) of PC/CL was dried in test tubes to produce the respective lipid films. In the following, 2 ml (1.2 mg ml^−1^) of an aqueous suspension of nanoparticulated magnetite was dispersed by ultrasonication for 2 min at 189 W. The magnetite suspension was added to the lipid films, which were previously prepared. By using a vortex, the sample was then vigorously shaken in a test tube for 10 s followed by incubation in an ultrasound bath, at room temperature, for 30 min. The magnetoliposomes were separated from lipid vesicles that were not associated with the nanoparticles by using magnetic decantation twice. For the preparation of magnetite capped by CTAB associated with ferrocene (Fe_3_O_4_-CTAB-Fc), a powder mass of Fe_3_O_4_ nanoparticles was weighed and re-dispersed in a 1 mM CTAB solution for a final concentration of 1.2 mg ml^−1^ and submitted to vigorous stirring. Fe_3_O_4_-CTAB was separated from pure CTAB micelles by magnetic decantation. Ferrocene dissolved in DMSO was added to the Fe_3_O_4_-CTAB suspension and sonicated for a final concentration of 730 µM.

### 2.5. X-ray diffraction

Powder X-ray diffraction (XRD) measurements were recorded on a Bruker D8 diffractometer operating with Cu K*α* radiation (*λ* = 1.5418 Å), 40 kV and 100 mA. The powder diffraction patterns were registered in the range of 2*θ* = 10–80°, with a step scan of 0.02° and at a rate of 10 s per step.

### 2.6. Fourier transform infrared spectroscopy

Infrared spectroscopy was used in ATR mode (attenuated total reflection) using a Varian AIM-8800 coupled to the microscope. The suspensions of nanoparticles were dropped onto the diamond crystal. The samples were slowly dried by the presence of silica particles nearby to the crystal. The drying was accompanied by successive scans until a significant contribution of water had disappeared.

### 2.7. FESEM images and energy dispersive X-ray analysis

High magnification images were obtained by field emission scanning electron microscopy (FESEM—JEOL model JMS-6701F) with energy dispersive X-ray spectrometer attached for chemical characterization (EDX—Thermo Scientific, model NORAN System—Nano Trace detector). A small drop of solution was pipetted on a piece of the doped silicon substrate and left to dry for hours before the image acquisition. The acceleration voltage used for FESEM images was 5 kV and for EDX analysis was 10 kV.

### 2.8. Fluorescence microscopy

Phase-contrast and fluorescence images of GUVs were obtained using an inverted optical microscope Varian Cary Model Eclipse.

### 2.9. GUV luminescence

A capillary glass tube was loaded with GUVs that were associated with MC540 delivered by LUV-magnetite. The fluorescent dye was excited by a green laser (*λ* = 532 nm) from a fibre-coupled laser source (Thorlabs Inc., Newton, NJ, USA). The capillary tube was coupled to an optical fibre which leads the fluorescence intensity to a spectrometer (Red Tide USB 650, Ocean Optics) connected to a computer for recording spectra.

### 2.10. Magnetic properties

The magnetic hysteresis properties were determined at room temperature using a vibrating sample magnetometer (Quantum Design SQUID-VSM) with the inducing field sweeping from −1.0 T to 1.0 T.

### 2.11. Magneto-switchable electrochemistry

Cyclic voltammetry experiments were carried out in a Metrohm Autolab potentiostat/galvanostat. Magnetophoresis of CTAB-magnetite was performed using a neodymium-iron-boron magnet perpendicularly positioned to the working electrode. The maximal magnetic field is 0.4 T. The electrochemical experiments were performed using a conventional electrochemical cell configuration. The electrochemistry was carried out with electrodes: glassy carbon as the working electrode (1.0 cm^2^), platinum as a counter electrode (1.5 cm^3^) and Ag/AgCl/Sat. KCl, as a reference electrode. It is important to mention that during the electrochemical experiments two configurations were used: ‘switch on’ and ‘switch off.’ The ‘off’ mode only corresponds to the suspension electrochemistry without the application of external magnetic field (without magnet). In the ‘on’ mode, the magnet was positioned behind the working electrode until the entire magnetic system was attracted.

### 2.12. Zeta potential and dynamic light scattering

Analysis of the zeta potential (*ζ*) for the formulations was obtained using the equipment Zetasizer^®^ Nano ZS (Malvern Instruments). The potential values were calculated as the average electrophoretic mobility values using the Smoluchowski equation [31]. Dynamic light scattering (DLS) measurements were performed using an ALV/CGS-3 compact goniometer system consisting of a 22 mW HeNe linearly polarized laser operating at a wavelength of 633 nm, an ALV 7004 digital correlator and a pair of avalanche photodiodes operating in the pseudo-cross-correlation mode. The samples were placed in 10 mm diameter glass cells and maintained at a constant temperature of 25 ± 1°C. The autocorrelation functions reported are based on three independent runs of 60 s counting time. The data were collected and further averaged by using the ALV Correlator Control software.

## 3. Results and discussion

### 3.1. PC/CL-capped and stabilized magnetite nanoparticles

**Figure 2.**
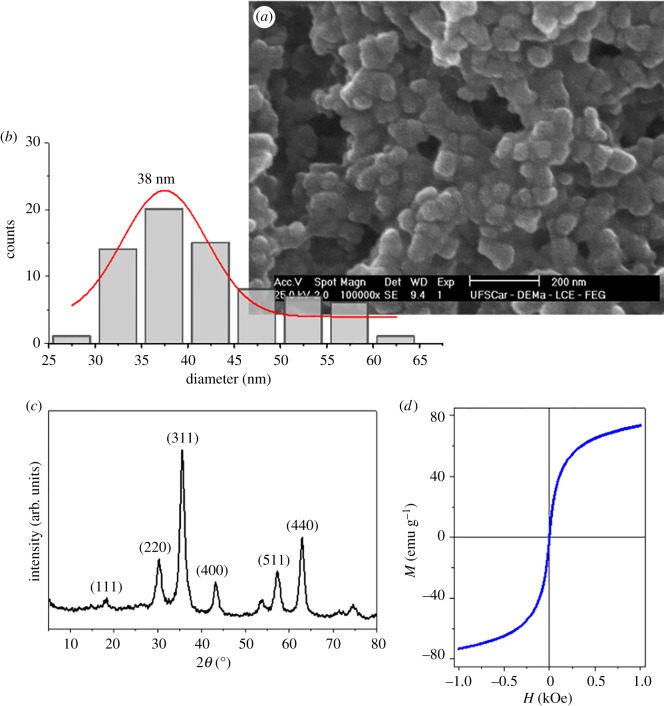
Characterization of synthesized magnetite nanoparticles. (*a*) Representative SEM image, (*b*) particle size distribution, (*c*) XRD pattern and (*d*) magnetization curve.

**Figure 3.**
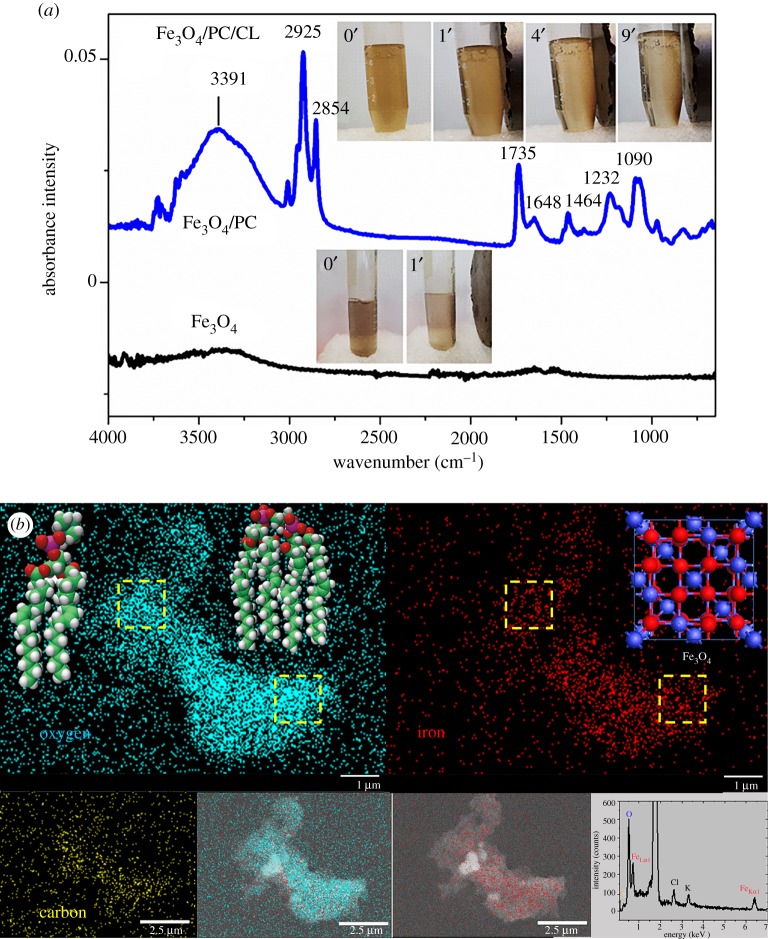
Analysis of Fe_3_O_4_/PC/CL composition. (*a*) FTIR spectra of bare and LUV-encapsulated magnetite nanoparticles. The black line corresponds to the spectrum of bare magnetite, the blue line to PC/CL-encapsulated magnetite. The insets show snapshots of temporal movement of magnetite in aqueous suspension (lower insets) and lipid-coated magnetite (upper insets) under the action of a magnet. The encapsulation by lipids delays the movement of the nanoparticles. The times are indicated in the snapshots. (*b*) FESEM images and energy dispersive X-ray spectroscopy (EDX) maps/spectrum. The elemental maps of oxygen (blue), iron (red) and carbon (yellow) are indicated in the respective panels. In two areas of the oxygen and iron maps, which are delimited by the yellow dashed squares, the higher oxygen density relative to that of iron is consistent with the contribution of oxygen in magnetite nanocrystals (crystalline structure at the right side of the figure) and POPC and TOCL (three-dimensional structures at the left side of the figure) that as expected should occupy a higher area than magnetite. The higher density areas of carbon signal are coincident with those of oxygen and iron. Concerning EDX limitation for the detection of low atomic number elements, the comparison of the carbon signal with the signals of oxygen and iron is merely qualitative. The smooth, bright areas of the image correspond to crystallization of KCl. The K^+^ and Cl^−^ are the counter ions of ammonium quaternary and phosphate groups of PC structure that crystallizes when the sample is dried for analysis. The acceleration voltage used for EDX analysis was 10 kV.

The scheme depicted in figure 1 shows the use of magnetite for signal transmission. Therefore, magnetite nanoparticles were synthesized as described in the Material and methods section and characterized by FESEM, XRD and SQUID-VSM magnetometer system (figure 2*a–d*). The magnetite nanoparticles synthesized are spherical with a mean size of 38 ± 4 nm. The agglomeration of the magnetite nanoparticles results from their high surface energy. Figure 2*c* shows the X-ray diffraction pattern of the magnetite nanoparticles sample. The XRD patterns confirmed that magnetite has formed under the conditions applied. The Bragg reflection peaks are (1 1 1), (2 2 0), (3 1 1), (4 0 0), (5 1 1) and (4 4 0) and the positions and relative intensities can be indexed to a face-centred cubic structure of magnetite with lattice parameters *a* = 8.42 ± 0.20 Å (JCPDS 85-1436), *a* = 8.40 Å [32]. The magnetic properties of magnetite nanoparticles were analysed by SQUID-VSM at room temperature (figure 2*d*). The reversible hysteresis curve (coercivity Hc = 0) is observed in figure 3 and indicates the superparamagnetic nature of the nanoparticles. The characteristics of superparamagnetism mean that this sample can be magnetized under the effect of an external magnetic field. The increase in the external magnetic field strength leads to the saturation of the magnetic moment (M) of magnetite nanoparticles.

However, without an external magnetic field, there is no remnant magnetic moment, i.e. the particle had the net moment randomized to zero. In the superparamagnetic regime, the nanoparticles become magnetic in the presence of an applied external magnetic field but revert to a non-magnetic state when the magnetic field is removed. This behaviour is necessary when these nanoparticles are introduced into living systems because, once the external magnetic field is removed, the magnetization disappears (due to negligible remnant magnetization and coercivity) and the agglomeration of nanoparticles is avoided [33]. Therefore, the superparamagnetic nanoparticles are very useful for biotechnological purposes as they are not subject to strong magnetic interactions in solution and are readily stabilized in physiological conditions. Figure 2*d* shows that the magnetic moment of this magnetite sample is saturated at approximately 72 emu g^−1^ with the external field of 1 T. The magnetic moment observed for magnetite is consistent with a nanoscale magnetic material that is lower than the corresponding bulk magnetic material (Ms(Fe_3_O_4_) = 90 emu g^−1^ at 298 K) [33–35]. The Fe_3_O_4_/PC/CL system presented a lower magnetization value due to the coating layer. According to the proposal of figure 1, the following step was the encapsulation of magnetite in LUVs as described in the Material and methods section.

PC/CL liposomes were prepared in the presence of Fe_3_O_4_ nanoparticles to result in magnetic particles inside the aqueous inner phase of liposomes. The magnetite-containing PC/CL liposomes were analysed by zeta potential and DLS and the values of surface charge and diameter are presented in table 1. The *ζ* value determined for bare magnetite (−30.1 ± 0.7 mV) is according to literature data, showing that the magnetite surface has a negative charge at neutral and alkaline pH [36,37]. After hydration of magnetite in the presence of lipids, the *ζ* values found were −24.3 ± 0.6 mV (75%) and −54.5 ± 1.2 mV. The value of −24.3 mV is consistent with PC/CL-covered magnetite nanoparticles. The value of −54.5 mV is consistent with magnetite-free liposomes because it is similar to the value obtained for PC/CL liposomes (−55 mV). The mean diameter of Fe_3_O_4_ (91 ± 5 nm) obtained by DLS is consistent with an aggregation of around two nanoparticles. The higher value of diameter (285 ± 11 nm) obtained when hydration occurs in the presence of Fe_3_O_4_ might result from variation of liquid surface tension upon the inclusion of nanoparticles at a high concentration of 1.2 g l^−1^. The value of 285 nm is the weighted average of a population of 350 nm that was 75% of the total nanostructures and a population of 90 nm that was 25% of the total nanostructures. Considering the delivery capacity of PC/CL demonstrated below, and the percentage of nanostructures with a mean diameter of 350 nm was the same that exhibited *ζ* values = −24.3 ± 0.6, encapsulated volume should be present. There is ambiguity in the literature concerning the term magnetoliposomes. In some cases, the term refers to liposomes (such as GUVs, LUVs) that encapsulated magnetic nanoparticles and in others, it refers to magnetic nanoparticles coated with lipid [38,39]. In this, we considered the data are consistent with the encapsulated magnetite inside liposomes.

**Table 1.** Zeta potential of bare and LUV-encapsulated magnetite nanoparticles.

sample	diameter (nm)^a^	*ζ* (mV)
Fe_3_O_4_	91 ± 5	−30.1 ± 0.7
PC/CL	144 ± 7	−55.7 ± 1.3
Fe_3_O_4_/PC/CL	285 ± 11	−24.3 ± 0.6 and −54.5 ± 1.2

^a^Weighted average values obtained by dynamic light scattering.

The presence of lipids covering magnetite nanoparticles was also corroborated by Fourier-transform infrared (FTIR) spectroscopy (figure 3*a*) of Fe_3_O_4_/PC/CL separated from the solution using a magnet. The FTIR spectra of magnetite nanoparticles presented vibration modes that were not present in the bare magnetic particles. The LUV-encapsulated magnetite has adsorbed water that is characterized by bands at 3342 and 3391 cm^−1^, which are attributed to *ν*O-H stretching vibration and HOH modes. The spectra of PC/CL-encapsulated magnetite presented bands peaking at 2925 and 2854 cm^−1^, which are assigned to the symmetric and asymmetric vibration of methylene (-CH_2_-) and methyl (-CH_3_). These vibration modes are consistent with the contribution of acyl chains of the phospholipids. Carbonyl (C = O) vibrations around 1735 cm^−1^ and quaternary ammonium (CN^+^−(CH_3_)_3_) asymmetric vibrations at 970 cm^−1^ were also observed for Fe_3_O_4_/PC/CL. The encapsulation of magnetite by phospholipids was also corroborated by the bands peaking at 1090 and 1232 cm^−1^ assigned to the vibrational modes for PO_4_^−^ [40]. Also, in figure 3*a*, the insets show snapshots of temporal movement of magnetite in aqueous suspension (lower insets) and lipid-coated magnetite (upper insets) under the action of a magnet. The times indicated in the snapshots show that the encapsulation by lipids delays the movement of the nanoparticles. All the characteristic absorption peaks in the spectra are consistent with magnetite encapsulation by phospholipids. The encapsulation of magnetite nanoparticles was also analysed by FESEM EDX technique (figure 3*b*). Figure 3*b* shows the elemental maps of oxygen (blue), iron (red) and carbon (yellow) as indicated in the respective panels. In two areas of the oxygen and iron maps, which are delimited by the yellow dashed squares, the higher oxygen density relative to that of iron is consistent with the contribution of oxygen in magnetite nanocrystals (crystalline structure at the right side of the figure) and POPC and TOCL (three-dimensional structures at the left side of the figure). In magnetite-encapsulated LUVs, it is expected that the lipids occupy a higher area than magnetite. The higher density areas of carbon signal are coincident with those of oxygen and iron. Concerning EDX limitation for the detection of low atomic number elements, the comparison of the carbon signal with the signals of oxygen and iron is merely qualitative. Maps of iron and oxygen signals overlapped with FESEM images and the EDX spectrum of the chemical elements are the lower panels at the side of carbon map. High-resolution FESEM image is shown in electronic supplementary material, figure S1.

### 3.2. Interaction of PC/CL liposomes with GUVs

**Figure 4.**
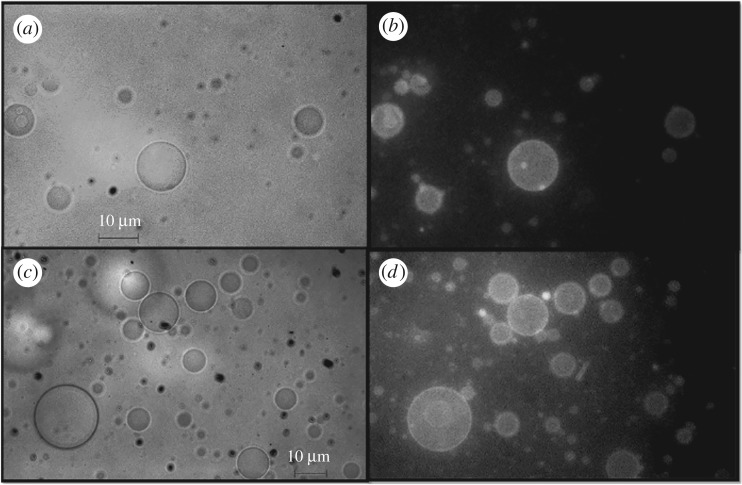
Fusion of LUVs with GUVs detected by fluorescence. (*a*) and (*b*) are, respectively, the phase-contrast and fluorescence images of GUVs devoid of fluorescent dyes after fusion with LUVs carrying MC540. (*c*) and (*d*) are, respectively, the phase-contrast and fluorescence images of GUVs devoid of fluorescent dyes after fusion with LUVs carrying MC540 and magnetite.

According to figure 1, nanostructured magnetite should be encapsulated by LUVs associated with a fluorescent dye and delivered to GUVs. To facilitate the fusion of the vesicles, GUVs were formed by a mixture of PC/PE/CL (50/30/20%, by weight, respectively). PC/CL LUVs were added to suspensions of GUVs, and the fusion of small with giant vesicles was analysed by the delivery of the fluorescent probe MC540 to GUVs. Figure 4*a,b* shows the images of fluorescent GUVs that exhibited this property after addition of LUVs/MC540. The experiment was repeated replacing PC/CL liposomes associated with MC540 by magnetite-containing PC/CL liposomes that were also associated with MC540 (figure 4*c,d*). In the latter condition, non-fluorescent GUVs became equally fluorescent suggesting that magnetite nanoparticles were delivered to GUVs as successfully as MC540 if the interior aqueous phase of the LUVs does not leak. Therefore, the successful delivery of magnetite nanoparticles by LUVs was tested by checking whether GUVs acquired the capacity to undergo deformation or magnetophoresis by the action of an external magnetic field, as described below.

### 3.3. Delivery of magnetic nanoparticles to GUVs

**Figure 5.**
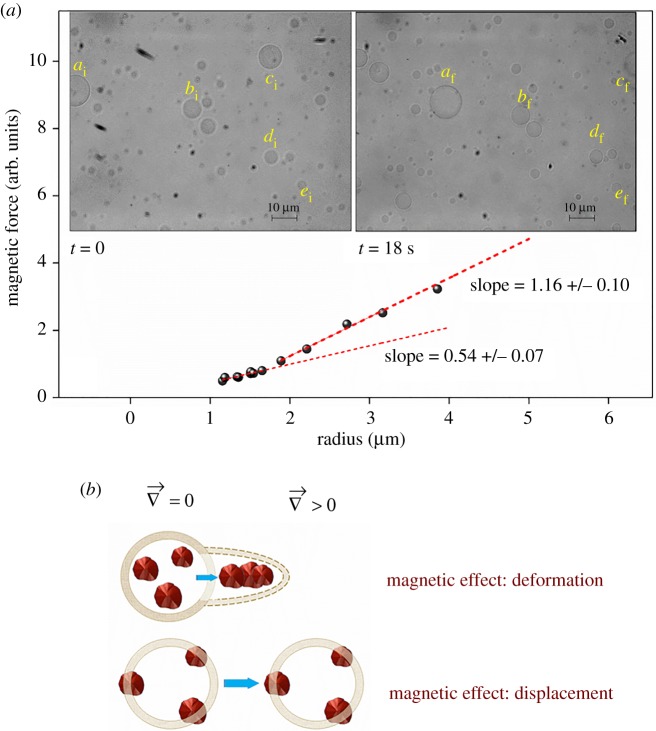
Optical microscopy images showing magnetophoresis of GUVs fused with LUVs loaded with magnetite in a condition like that described in figure 4. (*a*) Five GUVs are identified by the letters a, b, c, d and e followed by the subscript indices *i* (initial position) and *f* (final position) in time-lapsed images. The plot of magnetic force which is proportional to drag force versus GUV radius (blue points) was linearly fitted at two data intervals revealing different slopes for the GUV population with radii of 1.1–1.6 µm and 2.0–4.0 µm. (*b*) is an out-of-scale cartoon representing GUV magnetophoresis promoted by magnetite attached inside the lipid bilayers observed in (*a*) and GUV deformation by internal free magnetite under the action of a magnetic force that was not observed in the present study.

Considering that the viscous drag and the magnetic forces are the only forces acting on the particle, a balance of forces is required by the condition of the dynamic equilibrium [41]. Here, the term magnetophoresis or magnetic mobility is used to describe the movement of a superparamagnetic particle under the action of the magnetic force produced by a neodymium magnet. Figure 5 shows the result of a magnetophoretic effect on GUVs during the application of an external 0.4 T magnetic field provided by a magnet on the side of the microscopy lamina. The time interval between the two snapshots was 18 s (inset of figure 5*a*). The snapshots show that GUVs were attracted by the neodymium magnet that is consistent with the efficient delivery of the magnetic nanoparticles from LUVs to GUVs. The addition of bare magnetite to GUV suspension did not give to these vesicles the property to be moved by the magnetic force provided by a neodymium magnet (electronic supplementary material, figure S2). Stoke's equation gives the relationship between the drag force and the size and velocity of GUVs. At low Reynolds number, which is typical for the displacement of microscopic objects in fluids, the viscous drag forces dominate the inertial forces, and each single GUV offers a convenient way to measure the magnetic forces [41]. Therefore, the resulting drag force must be equal in magnitude to the magnetic effect for each GUV [42]. Identifying the radius and velocity of each GUV from the two images of figure 5*a*, it is possible to determine the magnetic force, up to an unknown factor that depends on the effective dynamic viscosity, given by equation (3.1).

3.1 $$F_{D}=6\pi \eta Ru,$$

where *η* is the effective dynamic viscosity of the fluid (Pa s), *R*, the radius (µm) and *u*, the relative velocity between the fluid and the GUV (µm s^−1^).

According to equation (3.1), a linear correlation of the magnetic force versus the GUV radius is expected.

However, figure 5*a* indicates an increased slope for larger GUV radius. An increased slope according to equation (3.1) indicates a larger velocity, which sounds counterintuitive, if one assumes that the magnetite content inside different-sized GUVs is unchanged. However, it is expected that larger GUVs could be loaded with a higher amount of magnetite, therefore increasing the effective magnetic moment of the bigger GUVs. As a result, a higher magnetic attraction is experienced under the external gradient of the magnetic field by the larger GUVs with a corresponding higher drag force. This increased drag force results in an increased velocity for bigger GUVs as indicated by the experimental data in figure 5*a*.

It is noteworthy that magnetite-loaded GUVs experienced displacement in a viscous medium under the influence of the neodymium magnet rather than any notable membrane deformation as previously reported. Shape deformation and membrane perturbation could result from the occurrence of aggregates of magnetite aligned under the action of the magnetic field [39]. However, GUVs deformed by the action of a magnetic field on internal magnetite can recover its initial curvature by pushing the cluster toward the inner lipid leaflet [43]. In the conditions of the present study, the absence of GUV deformation is suggestive of magnetite bound into the lipid bilayer as illustrated by figure 5*b*. Considering the thickness of the hydrophobic core of the bilayer is about 4 nm, only a small part of the magnetic particles may be inserted in the bilayer [44]. However, it is likely that these particles retain a layer of phospholipids that help their attachment to the membrane.

### 3.4. Applications of magnetic mobility

**Figure 6.**
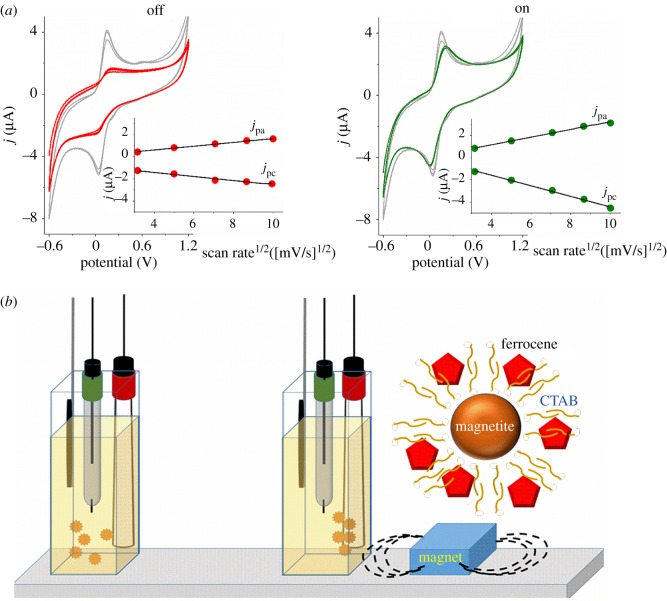
Magneto electrochemistry of Fe_3_O_4_-CTAB-Fc. The electroactivity was determined in the ‘switch off’ (red line) ‘switch on’ (green line) modes in the configuration I of Melo *et al.* [45]. (*a*) Cyclic voltammograms of Fe_3_O_4_-CTAB-Fc at 100 mV s^−1^ obtained at ‘switch on’ (green line) and ‘switch off’ modes (black line). The anodic (*j*_pa_) and cathodic (*j*_pc_) peaks as a function of the scan rates^1/2^ obtained in the ‘switch off’ (red points) and ‘switch on’ (green points) modes, respectively. The grey line corresponds to the voltammogram of ferrocene associated with CTAB micelles (control). Support electrolyte: 0.1 mol l^−1^ KCl, pH 7.0. (*b*) A possible organization of the supramolecular material Fe_3_O_4_-CTAB-Fc is shown in the scheme of the magneto electrochemistry configuration.

Figure 1 shows two possible implementations of magnetic liposomes: transmission of electric and luminous signals by magneto electrochemistry and spectrum transmission, respectively. Magnetophoresis of magnetic nano and microparticles has been used in magneto electrochemistry. In magneto electrochemistry, the catalysts and redox mediators immobilized on magnetite particles can be carried by magnetic fields to the surface of an electrode making feasible a switchable control of faradaic current from catalysis. As an example, Melo *et al.* synthesized of a new material composed by magnetite modified with insoluble ferrocene and chitosan cross-linked with glucose oxidase (Fe_3_O_4_-Chi-Fc/GOx) [45]. The authors observed an increase of 70% of the electrical current generated by the catalysis of GOx. In this, for the application of magnetophoresis in cyclic voltammetry, a similar experiment was carried out using magnetite capped by CTAB associated with ferrocene (Fe_3_O_4_-CTAB-Fc, sketched in figure 6). The supramolecular system Fe_3_O_4_-CTAB-Fc was characterized by zeta potential. The efficient covering of magnetite by CTAB was corroborated by the drastic change of the zeta potential from −32 to + 58 mV. The electrochemical behaviour of 1.2 mg ml^−1^ of Fe_3_O_4_-CTAB-Fc was determined in the presence and absence of an external magnetic field. Magneto electrochemistry was carried out using configuration I described by Melo *et al.* in which ‘switch off’ condition corresponds to the measurement carried out at zero magnetic fields. Figure 6*a* shows the cyclic voltammograms of the Fe_3_O_4_-CTAB-Fc obtained at 100 mV s^−1^ using ‘switch off’ (red line) and ‘switch on’ (green line) modes, respectively. Also, in figure 6, the grey line corresponds to the voltammogram of ferrocene associated with CTAB micelles (control condition). The insets show the anodic (*j*_pa_) and cathodic (*j*_pc_) peaks as a function of the scan rates^1/2^ obtained in the ‘switch off’ (red points) and ‘switch on’ (green points) modes, respectively. A possible organization of Fe_3_O_4_-CTAB-Fc is shown in the scheme of the magneto electrochemistry configuration (figure 6*b*). At 100 mV s^−1^ scan rate, the ‘switch on’ mode increased approximately 45% the values of current intensity for the anodic and cathodic peaks. The Fe_3_O_4_-CTAB-Fc is attracted by the external magnetic field and concentrated around the electrode in a condition of the applied magnetic field. Therefore, the magnetic field helps the collision of Fe_3_O_4_-CTAB-Fc particles with the electrode and increases the current intensity. The external magnetic field is applied 1 min before the starting of the cyclic voltammetry measurement. Similarly to the observed for Fe_3_O_4_-Chi-Fc/GOx, ferrocene associated with CTAB capping magnetite also exhibited a quasi-reversible redox process (Δ*E*_p_ = *E*_pa_ − *E*_pc_ higher than 0.059 V) with *E*_pa_ and *E*_pc_ signals of 0.15 V and 0.034 V, respectively. Bared Fe_3_O_4_ particles did not show electroactivity, and CTAB in the magnetite-free system (grey line) exhibited very low *E*_pa_ and *E*_pc_ signals of 0.62 and 0.21 V, respectively.

The CTAB-Fc electroactivity was abolished in the system containing magnetite (red and green lines). In the ‘switch on’ mode, Fe_3_O_4_-CTAB-Fc particles showed *E*_pa_ = 0.21 V and *E*_pc_ = 0.00 V that results in Δ*E* switch on = 0.21 V at 100 mV s^−1^. In the ‘switch off’ mode, Fe_3_O_4_-CTAB-Fc particles showed *E*_pa_ = 0.18 V and *E*_pc_ = 0.02 V that results in *ΔE* switch off = 0.16 V.

**Figure 7.**
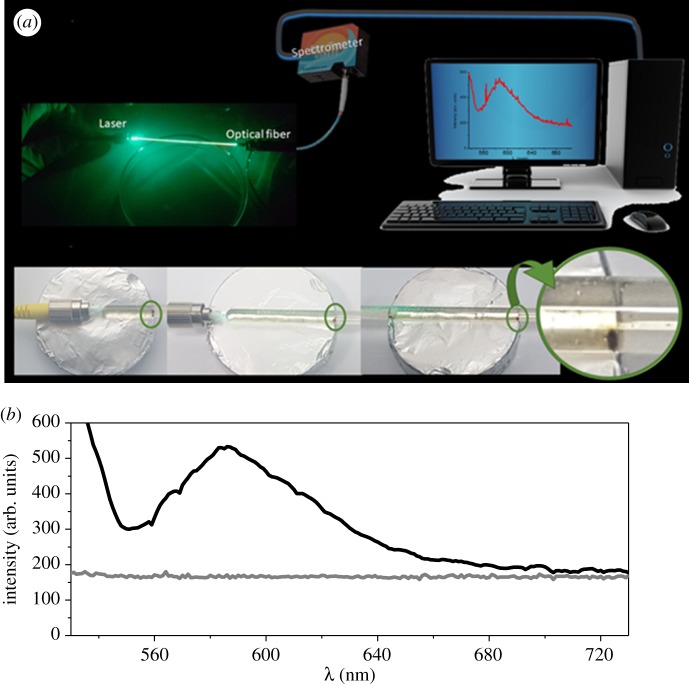
The switchable light signals by magnetophoresis. (*a*) shows the fluorescence of GUVs that were loaded with MC540 and magnetite and had magnetophoretic properties. The capillary tube containing GUVs is in close contact with an optical fibre delivering the excitation laser. The fluorescence emission spectra of GUVs were transmitted by the optical fibre, and the spectrum could be recorded as shown in (*b*). In (*b*), the grey line corresponds to the signal of non-fluorescent GUVs and the black line to the spectrum of MC540 associated with GUVs.

Also, luminous signals can become switchable by magnetophoresis. In this set of experiments, magnetite-loaded LUVs associated with MC540 were used to deliver magnetite and the fluorophore to GUVs as described before. The suspension, i.e. magnetite-loaded GUVs, was transferred to a similar capillary tube that had the extremities coupled to a laser source and an optical fibre connected to a fluorometer (figure 7*a,b*). GUVs loaded with magnetic nanoparticles are susceptible to magnetophoresis as previously demonstrated. The fluorometer could record the fluorescence emitted by magnetite-loaded GUVs. Figure 7*a* shows a set-up of the experiment. The green laser is applied to the capillary that is connected to an optical fibre that in turn is connected to the fluorescence spectrometer. The signal captured by the spectrometer is transmitted to the computer. Figure 7*a* also shows the snapshots of magnetite-loaded GUVs moved along the capillary by using a magnet. Figure 7*b* shows the fluorescence spectrum of MC540 associated with magnetite-loaded GUVs that was recorded by the spectrometer.

In summary, the use of magnetite-containing liposomes for controlled transmission of signals constitutes a new glance on a system conventionally used for drug delivery, sensing and catalysis. It was demonstrated that fluorescent GUVs could transmit the luminescent signal using an optical fibre. Likewise, when a zwitterionic GUV acquires a positive or negative net charge due to fusion with a positively or negatively charged LUV, it can be electrophoretically moved, and when loaded with magnetite it can experience magnetophoresis. In addition to the fundamental features of magnetite-containing liposomes for directing the transmission of signals, we glimpse other applications, including the fabrication of chemical systems that can be used to execute Boolean logic operations. For instance, in a recent study [20], Liu *et al.* presented a system with gold nanoparticles coated by a photosensitive molecule that isomerizes under UV light, gains affinity for copper ions and promote nanoparticles aggregation leading to colour suspension change from red to violet. In that system, the authors have a binary code 0–0 when the dispersed gold nanoparticles coated by the photosensitive molecule are in a solution free of copper ions and not under UV irradiation. The 1–0 code is represented by the dispersed nanoparticles coated by the UV-isomerized molecules in the absence of copper ions. The code 0–1 is represented by dispersed nanoparticles coated by non-isomerized molecules (UV light absent) in the presence of copper ions. The code 1–1 is represented by the simultaneous presence of UV irradiation and copper ions that link and aggregate gold nanoparticles by traversed binding to UV-isomerized molecules. Based on this recent finds [20], we speculate that the system described here can be applied similarly. LUVs without magnetite and a fluorophore added to a suspension of GUVs constitute the 0–0 code since fusion occurs, but this is not detectable by the fact that GUVs do not gain fluorescence and magnetophoretic property. LUVs associated with a fluorophore, but without magnetite, make the GUVs fluorescent but the signal cannot be transmitted at a distance, and this condition represents the 1–0 code. The fluorophore-free LUVs loaded with magnetite fuse with the GUVs and give them the magnetophoretic property but not luminous signal to be transmitted, and this condition constitutes the code 0-1. Only LUVs with fluorophore and magnetite rendered GUVs fluorescent and owned of magnetophoretic property, and this condition represents the code 1–1.

## 4. Conclusion

We demonstrated that LUVs with different compositions could fuse with GUVs and act as a delivery system to modify GUV properties. Bare magnetite (Fe_3_O_4_) nanoparticles align and undergo magnetophoresis when submitted to an external magnetic field, but they are not able to penetrate in the GUV membranes by the collision. However, magnetic nanoparticles were stabilized and covered by phospholipids composing LUVs and, in this condition, they were efficiently delivered to GUVs. GUVs loaded with magnetite are susceptible to the action of an external magnetic field of 0.4 T. GUV also became fluorescent when fused with LUVs loaded with the fluorescent dye MC540. Although GUVs are commonly seen as a model of the plasma membrane of cells, the modification of the electrochemical, photophysical and magnetic properties of GUVs by the fusion with LUVs points to a diversity of applications for this system. Finally, we discussed possible applications, including a variety of sensors for biological systems, therapeutic applications, signal transduction and bio-computing.

## Supplementary Material

Supplemental Material

## Data accessibility

The datasets supporting this article have been uploaded as part of the electronic supplementary material.
