# Supplementary material for: Magnetoliposomes as model for signal transmission
Source: R Soc Open Sci. 2019 Jan 16;6(1):181108. doi: 10.1098/rsos.181108 (PMC6366231; doi:10.1098/rsos.181108)
Supplement: Supplemental Material [file rsos181108supp1.docx]

Magnetoliposomes as Model for Signal Transmission

**G. R. Barreto^1^, C. Kawai^1^, A. Tofanello^1^, A. A. R. Neves^1^, J. C. Araujo-Chaves^1^, E. Belleti^1^, A. J. C. Lanfredi^2^, F. N. Crespilho^*3^ and I. L. Nantes-Cardoso^*1^**

*1.* *Center of Natural Sciences and Humanitie (CCNH), Federal University of ABC (UFABC), Santo André, SP, Brazil.*

*2.* *Center for Engineering and Applied Social Sciences (CECS), Federal University of ABC (UFABC), Santo André, SP, Brazil.*

*3. São Carlos Institute of Chemistry, University of São Paulo (USP), Av. Trabalhador São-carlense, 400, São Carlos, São Paulo 13560-970, Brazil.*

.

**Keywords:** Giant unilamellar vesicles, large unilamellar vesicles, nanoparticulated magnetite, signal transmission.

*Author for correspondence [ilnantes@ufabc.edu.br](mailto:ilnantes@ufabc.edu.br), [frankcrespilho@iqsc.usp.br](mailto:frankcrespilho@iqsc.usp.br)).

1. Supplemental Results

Figure 1S shows GUVs in the presence of bare magnetite added to the suspension, during the application of an external 0.4T magnetic field provided by a neodymium magnet on the side of the microscopy lamina. The time interval between the two snapshots is 3 s. The snapshots show that bare magnetite aggregates moved attracted by the neodymium magnet. In this condition, GUVs did not incorporate bare magnetite and did not experience any significant displacement even when moving magnetite aggregates collided with them (see a zoom of the snapshots). Therefore, the addition of bare magnetite to GUV suspension did not give to these vesicles the property to be moved by the magnetic force provided by a neodymium magnet.


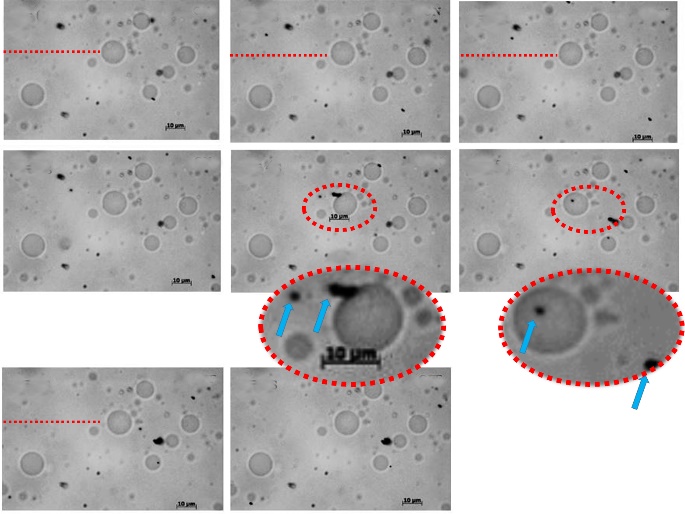


Figure 1S. Magnetic movement of bare magnetite in the presence of GUVs. Zoom images show that moving bare magnetite aggregates collide with GUVs leading to a discrete reversible vesicle deformation. The upper six panels are snapshots with around 3 s of time intervals in the presence of a neodymium magnet. The lower two panels are the snap shots obtained after the neodymium magnet is removed. In this condition, GUVs remained immobile.

Figure 2S shows the FESEM high resolution image of Fe_3_O_4_/PCCL that was used for EDX analysis in Figure 3B.


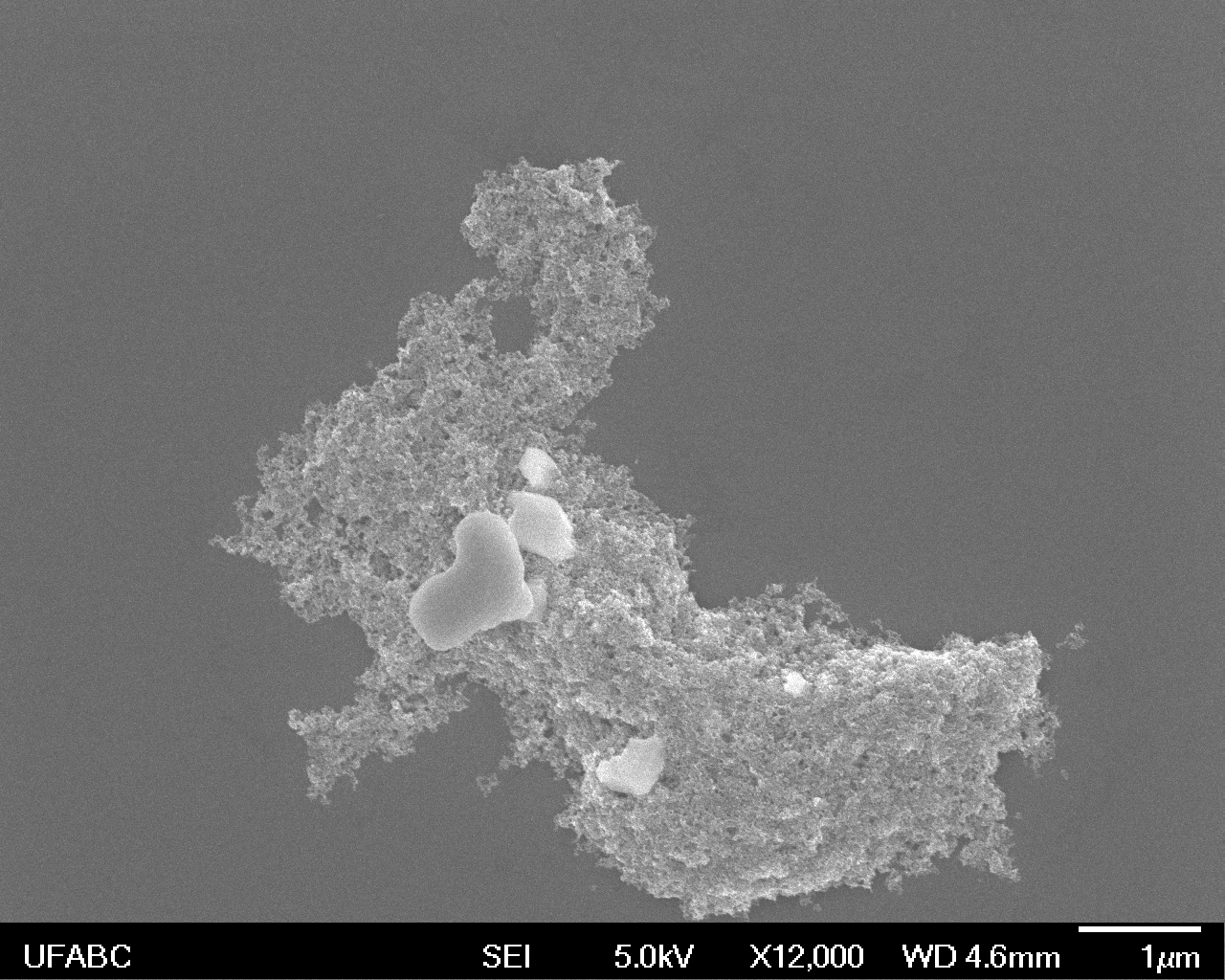


Figure 2S. High magnification image of Fe_3_O_4_/PCCL liposomes obtained by Field Emission Scanning Electron Microscopy (FESEM – JEOL model JMS-6701F). The acceleration voltage used for FESEM image was 5 kV. The smooth clear areas of the image correspond to crystallization of KCl. The K^+^ and Cl^-^ are the counter ions of ammonium quaternary and phosphate groups of PC structure that crystalize when the sample is dried for analysis.
